# Subharmonic injection-locked photonic integrated thin-film lithium niobate optoelectronic oscillator

**DOI:** 10.1515/nanoph-2025-0476

**Published:** 2025-11-19

**Authors:** Zijun Huang, Rui Ma, Qiang Ying, Peng Hao, Wei Ke, Xinlun Cai, X. Steve Yao

**Affiliations:** State Key Laboratory of Optoelectronic Materials and Technologies, School of Electronics and Information Technology, 26469 Sun Yat-Sen University, Guangzhou 510275, China; Photonics Information Innovation Center and Hebei Provincial Center for Optical Sensing, College of Physics Science and Technology, Hebei University, Baoding 071002, China; NuVison Photonics, Inc, Las Vegas, NV 89109, USA; Hefei National Laboratory, Hefei 230088, China

**Keywords:** integrated optoelectronic oscillator, subharmonic injection locking, thin-film lithium niobate, phase noise, high frequency

## Abstract

Integrated optoelectronic oscillators (OEOs) have emerged as pivotal enablers of compact, energy-efficient solutions for generating high-frequency radio-frequency (RF) signals with exceptional spectral purity – an essential demand in the advancement of radar and communication technologies. Yet, the quest for ultra-low phase noise near the carrier remains hampered by laser frequency instability and environmental fluctuations. In this work, we unveil the first photonic-integrated, high-order subharmonic injection-locked OEO realized on a thin-film lithium niobate (TFLN) platform, seamlessly uniting a Mach–Zehnder modulator (MZM) and an add-drop microring resonator (MRR) in a monolithic architecture. By harnessing an external RF source operating at a fractional subharmonic (1/2*N*, with *N* = 1, 3, 5…) of the OEO’s free-running frequency, our system achieves robust locking to the 2*N*-th harmonic of the injected signal, made possible through the beating of ±*N*-th order modulation sidebands – precisely selected by the dual resonances of the MRR – at the photodetector. We experimentally demonstrate the generation of 28.7 GHz signals via second- and sixth-order subharmonic injection locking, employing external RF injections at 14.35 GHz and 4.78 GHz, respectively. This yields an outstanding side-mode suppression ratio (SMSR) exceeding 78 dB and remarkably low spurious emissions. Furthermore, the measured phase noise achieves values below −80 dBc/Hz at 100 Hz and below −115 dBc/Hz at 10 kHz offsets from the 28.7 GHz carrier, delineating a new standard for integrated OEO performance.

## 1 Introduction

Radar and communication systems require compact, reliable RF sources with low phase noise [1], [2], [3]. Integrated optoelectronic oscillators (OEOs) [4], [5], [6], [7], [8], [9], [10] generate high-frequency, high-purity RF signals using low-loss optical fibers [11] or high-*Q* resonators [12]. While SOI and InP platforms have enabled several integrated OEOs [4], [5], [6], [7], they face material limitations such as nonlinear, absorptive, and temperature-sensitive EOMs [13], [14], [15], [16], restricting their RF output below Ka-band. Thin-film lithium niobate (TFLN) has emerged as a superior alternative due to its wide bandgap and strong electro-optic properties, enabling Ka-band signal generation [9], [10]. However, TFLN and hybrid SiN–LN OEOs still struggle with phase noise near the carrier, primarily caused by laser frequency jitter [17], [18], [19] and random mechanical vibrations [20], in addition to poor SMSR, and spurs, especially when long fibers are used to suppress phase noise.

Subharmonic injection locking addresses key challenges by injecting a low-frequency RF reference signal into the OEO to lock the oscillator to the reference, improving phase noise near the carrier, enhancing SMSR, and minimizing spurs. This method also lowers the frequency requirement for the injection source, eliminating the need for high-frequency references. Previous subharmonic injection-locked OEOs used discrete components and operated below 10 GHz [21], [22] but were bulky and costly. Integrating photonic components onto a single chip offers a more compact, affordable, and reliable solution. However, a photonic-integrated subharmonic injection-locked OEO has yet to be reported.

Here, we present the first subharmonic injection-locked photonic integrated OEO on the TFLN platform, incorporating a Mach–Zehnder modulator and a microring resonator (MRR). The OEO locks to the 2*N*-th (*N* = 1, 3, 5, …) harmonic of the injected RF signal, enabling both second- and sixth-order subharmonic injection locking. By injecting RF signal at 14.35 GHz or 4.78 GHz, we achieve a 28.7 GHz output with a SMSR over 78 dB, improving by more than 60 dB compared to a free-running OEO with 1 km single mode fiber (SMF). This method lowers the needed injection frequency to subharmonics of the free-running oscillation. Phase noise at 100 Hz offset is below −80 dBc/Hz, over 50 dB better than the free-running case, with improved SMSR and suppressed spurs.

## 2 Scheme and principle

**Figure 1:**
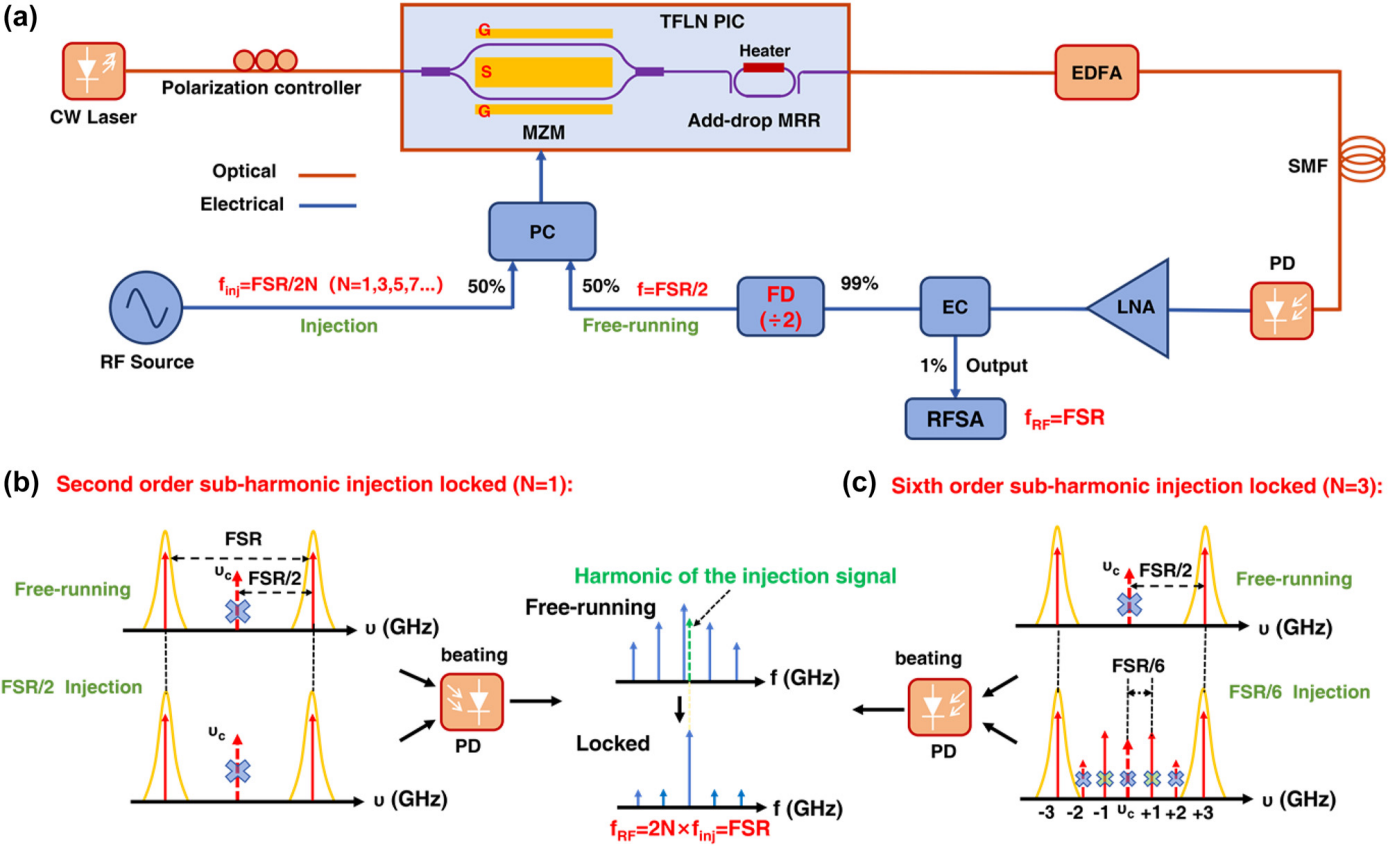
Subharmonic injection-locked OEO based on the TFLN platform. (a) Schematic. The principle of the second order (b) and the sixth order (c) subharmonic injection-locked OEO. PC: power coupler, FD: frequency divider, FSR: free spectral range, EC: electrical coupler, LNA: low noise amplifier, PD: photodetector, EDFA: erbium-doped fiber amplifier, MZM: Mach–Zehnder modulator, RFSA: RF spectrum analyzer.

Figure 1a presents the schematic of the subharmonic injection-locked OEO, which utilizes a TFLN photonic integrated chip (PIC) comprising an MZM and an add-drop MRR. The continuous wave (CW) optical carrier is set to the center frequency between two adjacent resonance peaks by thermally tuning the MRR. Prior to entering the PIC, the light passes through a polarization controller, ensuring alignment with the TE mode of the PIC. The MZM, biased at its zero-transmission point, effectively suppresses both the optical carrier and even-order modulation sidebands while retaining the odd-order sidebands. Serving as a comb filter, the add-drop MRR allows only those modulation sidebands that coincide with the two adjacent transmission peaks to pass through with minimal loss; these are subsequently amplified by an erbium-doped optical fiber amplifier (EDFA) to offset optical losses. A 1 km SMF with a typical dispersion value of 17 ps/(nm⋅km) at around the 1,550 nm wavelength is implemented to help reduce RF phase noise. Modulation sidebands selected by the adjacent resonance peaks are directed to the photodetector (PD), where they beat to produce an RF signal with a frequency equal to the free spectral range (FSR) of the MRR. In the traditional OEO schemes, the RF signal is generated by the beating of the optical carrier with the positive and negative *N*-th order modulation sidebands. The phase noise of this signal is significantly influenced by fiber dispersion, which typically requires dispersion compensation for long-delay fibers. However, in our work, the oscillation signal is directly derived from the beating of the two positive and negative *N*-th order modulation sidebands, which minimizes the impact of fiber dispersion on the phase noise of the RF signal. Therefore, a conventional SMF is utilized instead of dispersion-compensating fiber. The RF signal is then boosted by a low-noise amplifier (LNA). Ninety-nine percent of the RF output is routed via an electrical coupler (EC) to a frequency divider (FD) with a division ratio of 2, whereas one percent is sent to an RF spectrum analyzer (RFSA) for measurement. The feedback path divides the RF signal’s frequency by half using the FD, and this FSR/2 signal is fed back to the MZM through a power coupler (PC) to complete the OEO loop. Oscillation is initiated when the system gain exceeds unity [23].

To implement the subharmonic injection-locked OEO, an RF signal at a subharmonic frequency (FSR/2N, where *N* = 1, 3, 5, 7…) is injected into the free-running OEO loop via the PC’s second input. A beat signal is generated by beating the ±Nth-order modulation sidebands in the photodetector (PD), which will oscillate in the OEO loop, without involving the optical carrier. The locking range depends on the OEO’s mode spacing, set by the loop length, and the voltage ratio of the injected signal to the free-running mode [24], [25], [26]. Further details are provided in the Supplementary Files.

Figure 1b illustrates the mechanism of second-order subharmonic injection-locked OEO, in which an RF signal at FSR/2 is injected. With the MZM biased at zero transmission and the optical carrier thermally tuned to the midpoint between two adjacent MRR transmission peaks, the injected signal at FSR/2 generates an RF signal at the FSR, thereby locking the free-running OEO’s RF output of identical frequency. By adjusting the power of the injected signal to its optimal level, this approach combines the excellent frequency stability of the RF synthesizer with the exceptional phase noise performance inherent to OEOs. Furthermore, the selected mode gets additional gain from the injected RF signal, which ensures it wins mode competition against other modes for achieving a high SMSR.

Figure 1c presents the operating principle of a sixth-order subharmonic injection-locked OEO, which involves injecting an RF signal at a frequency of FSR/6 into the OEO. Following modulation by the MZM, both the optical carrier and ±second-order modulation sidebands are suppressed, while the ±first-order and ±third-order modulation sidebands remain. Among these, only the third-order modulation sidebands are selected by two adjacent resonance peaks of the MRR for transmission to the PD for beating. This injected signal at FSR/6 subsequently enhances the OEO oscillation at the FSR frequency, thereby locking the OEO oscillation to this injection frequency, ensuring the oscillation with low phase noise, high SMSR, and low spur.

## 3 PIC fabrication and characterization

**Figure 2:**
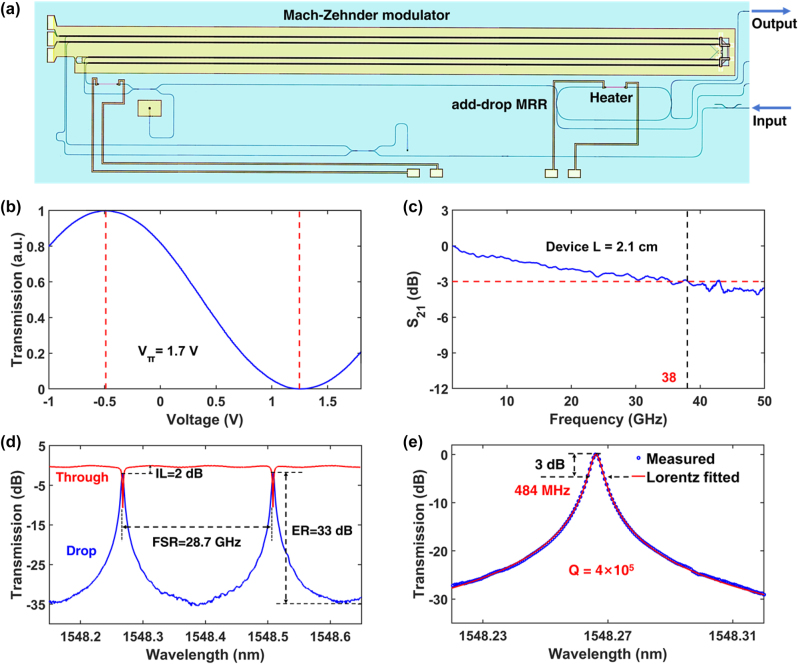
Characterization of the TFLN PIC. (a) The microscope photo of the TFLN PIC containing an MZM and add-drop MRR. (b) The measured normalized optical transmission spectrum of MZM as a function of the applied DC voltage, showing a *V*_*π*_ of 1.7 V. (c) The measured EO bandwidth (S_21_ parameter) of MZM with a total modulation length of 2.1 cm. (d) The measured transmission spectrum of the add-drop MRR showing the FSR, ER and IL. (e) The transmission spectrum of one of drop ports of the add-drop MRR showing the 3 dB bandwidth and *Q* value.

The PIC for the subharmonic injection-locked OEO is designed and fabricated on an X-cut TFLN platform, as depicted in Figure 2a. The detailed PIC fabrication process can be found in the materials and methods section. An MZM with low half-wave voltage (*V*_*π*_) and large electro-optic (EO) bandwidth is crucial for our device. The low *V*_*π*_ helps to produce the modulation sidebands with high power for reducing the required power of the external injection RF signal. The large 3 dB EO bandwidth ensures the OEO to generate the high frequency signals. The TFLN MZM is equipped with folded capacitance-loaded traveling-wave electrodes (CL-TWEs) with a total modulation length of 2.1 cm, to reduce the geometric length of the device while maintaining a low driving voltage. Figure 2b shows that the measured *V*_*π*_ is only 1.7 V, which is significantly lower compared to the typically *V*_*π*_ of >4 V with commercial MZMs fabricated with bulk LN [27], [28].

The measured 3 dB EO bandwidth is 38 GHz enabling the generation of high frequency signals at Ka-band, as shown in Figure 2c. The EO bandwidth only needs to support the oscillation frequency; it should not be too large, otherwise it will result in a large *V*_*π*_, generating low-power modulation sidebands and increasing the power requirement for the external injected RF signal.

The add-drop MRR is another key element for our device, which serves as optical bandpass filter for OEO single mode selection. The MRR should be featured with a narrow bandpass bandwidth, suitable FSR, and low insertion loss (IL) to generate a low phase noise high-frequency RF signal. The higher the *Q* factor, the better the selectivity of the filter [29], [30], [31]. The specific design parameters including the width of the waveguide and coupling spacing are almost the same as those of our previous designed MRR [10], which can be found in the materials and methods section. Figure 2d shows the measured transmission spectra of both the through (red curve) and drop (blue curve) ports of the add-drop MRR. The FSR is measured to be 28.7 GHz, corresponding to the circumference of 4.75 mm, for realizing 28.7 GHz OEO oscillation and filtering out other oscillation modes. The FSR does not need to be excessively large; it only needs to meet the requirements of the oscillation frequency. An overly large FSR would result in a smaller mode volume and a reduced *Q*-factor, thereby decreasing the mode-selectivity of the MRR filter. An extinction ratio (ER) of 33 dB and an IL of 2 dB are obtained. The measured 3 dB bandwidth of the drop port at a wavelength of 1,548.267 nm is 484 MHz, corresponding to a *Q* value of 4 × 10^5^, as shown in Figure 2e. Reducing the waveguide scattering loss caused by sidewall roughness can further increase the *Q* and minimize the IL.

## 4 Free-running OEO

The free-running OEO utilizing the described device is presented. An optical carrier at a wavelength of 1,550.14 nm and power of 18 dBm from the HAN’S RAYPRO laser is positioned between two adjacent transmission peaks of the MRR through thermal tuning. The MZM is set at the zero-transmission point to suppress the optical carrier and even-order modulation sidebands, resulting in the retention of odd-order modulation sidebands. Only the first-order modulation sidebands are selected by the transmission peaks of the MRR due to the relatively low power of higher-order odd-order modulation sidebands. The output power from the TFLN PIC is measured at −17 dBm. An EDFA (Amonics: AEDFA-PKT-DWDM-15-B-FA) with a gain of 17 dB compensates for optical loss, providing 0 dBm power to the PD. The RF signal is amplified by an LNA with a gain of 36 dB.

**Figure 3:**
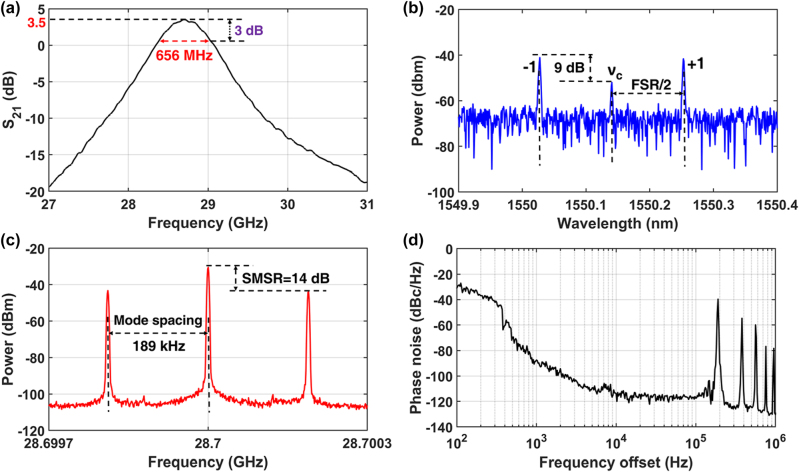
Free-running OEO. (a) The measured frequency response of the OEO’s SSOL gain consisting of the laser, the MZM, the add-drop MRR, the EDFA with a nominal gain of 17 dB, the PD, the LNA with a power gain of 36 dB, the EC, the FD (RF BAY FBS-2-40) with a divide ratio of 2, and the PC. (b) The measured optical spectrum. (c) The RF spectrum of the 28.7 GHz signal measured with an RFSA with a frequency span of 600 kHz and a RBW of 2.4 kHz. (d) The measured phase noise of the 28.7 GHz RF signal generated by the free-running OEO.

Figure 3a shows the measured frequency response of the small signal open loop (SSOL) gain, which is characterized by using a vector network analyzer (VNA) (Agilent Technologies: N5227A) to measure the transmission coefficient S_21_, with the experimental setup showing in the Supplementary Files. It has a center frequency of 28.7 GHz, a 3 dB bandwidth of 656 MHz, and a peak gain of 3.5 dB. The center frequency and bandwidth of the SSOL gain is determined by the FSR and the 3 dB bandwidth of the add-drop MRR, respectively. The slightly difference between the measured MRR’s bandwidth and the OEO’s SSOL bandwidth is due to that the optical carrier is not exactly at the center of two resonance peaks of the MRR. Figure 3b shows the measured optical spectrum measured with the optical spectrum analyzer (WaveAnalyzer 1500S). The optical carrier is not completely suppressed due to that the bias point of the MZM is not the ideal zero-transmission point. Although the optical carrier is residual, it cannot influence the RF signal as its power is 9 dB lower than that of the first-order modulation sidebands. Figure 3c is the RF spectrum of the OEO oscillation measured with a RF spectrum analyzer (RFSA, Agilent Technologies: PXA N9030A) operating at a frequency span of 600 kHz and a resolution bandwidth (RBW) of 2.4 kHz, showing the center oscillation frequency of 28.7 GHz, a side-mode spacing of 189 kHz, and a SMSR of 14 dB. As discussed previously, the 28.7 GHz is defined by the FSR of the MRR. The poor SMSR is due to a small side-mode spacing of 189 kHz determined by an OEO loop length of 1.058 km, which leads to a number of oscillation modes within the 3 dB bandwidth of 488 MHz of the MRR. Figure 3d shows the phase noise curve of the free-running OEO, measured with the RFSA (Agilent Technologies: PXA N9030A) having a phase noise measurement plug-in module. The phase noise of the free-running OEO at offsets below 400 Hz is poor, which is primarily attributed to the fluctuations of the fiber-chip coupling due to the random mechanical vibrations and the laser’s frequency jitter. Nevertheless, at frequency offsets above 400 Hz, the free-running OEO exhibits lower phase noise because of a 1 km-long SMF. At the frequency offsets at 100 Hz and 10 kHz, the phase noises for 28.7 the GHz oscillations are approximately −30 and −115 dBc/Hz, respectively. And at frequency offsets above 189 kHz, the phase noise significantly deteriorates due to the spurs at integer multiplies of 189 kHz, induced by a loop length of 1.058 km.

## 5 High-order subharmonic injection-locked OEO

**Figure 4:**
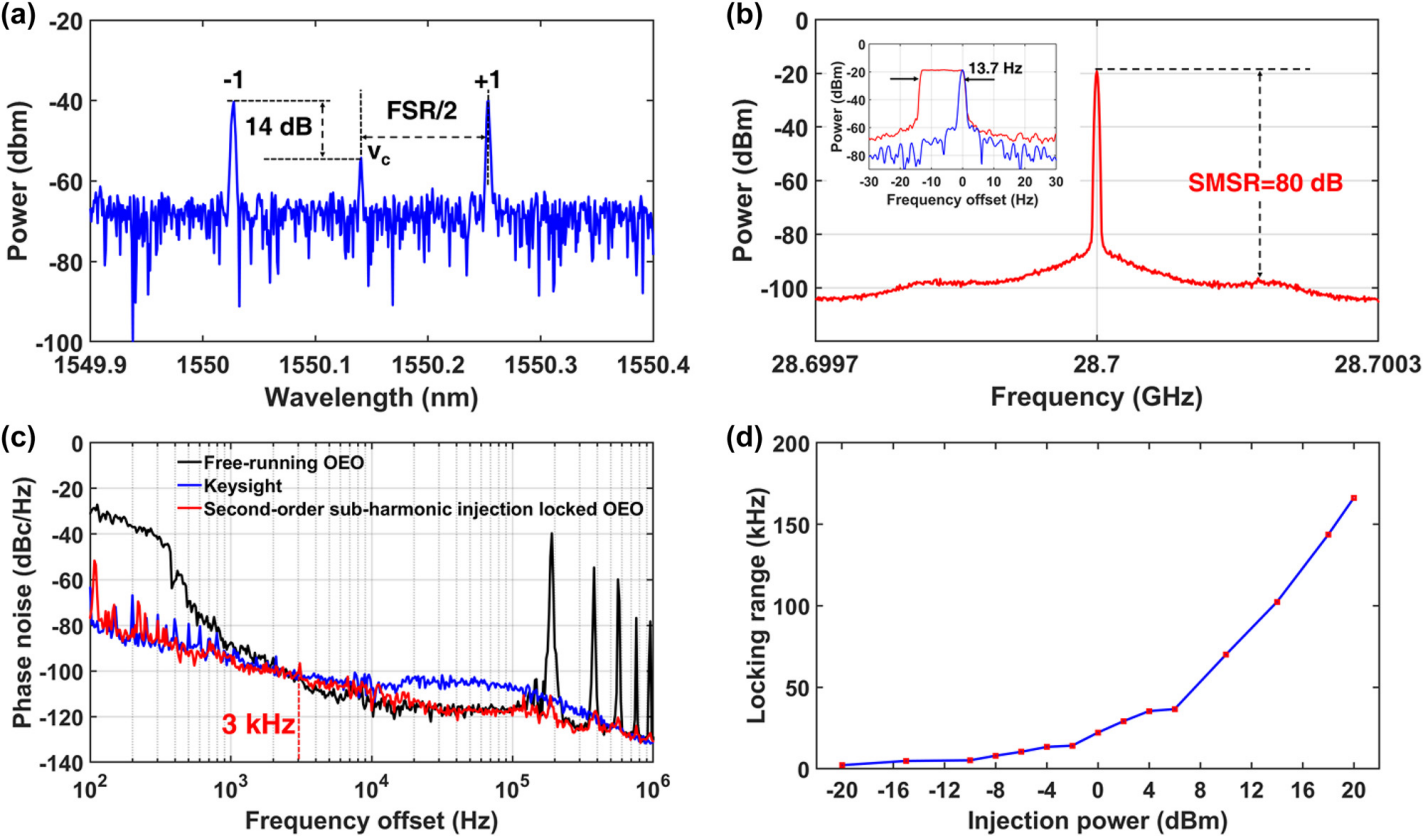
Second-order subharmonic injection-locked OEO. (a) The measured optical spectrum. (b) The RF spectrum of the 28.7 GHz signal measured with an RFSA with a frequency span of 600 kHz and a RBW of 2.4 kHz. Inset: the RF spectrum of the second-order subharmonic injection-locked OEO measured with an RFSA at Maxhold mode (blue curve) within 13 min and normal mode (red curve). (c) The measured phase noises of the 28.7 GHz RF signal generated by the free-running OEO (black curve), the second-order subharmonic injection-locked OEO (red curve), and the external injected RF source (Keysight E8257D) (blue curve). (d) The measured locking range as a function of the injection power.

The 28.7 GHz free-running optoelectronic oscillator (OEO) is subsequently injection locked using the second-order subharmonic of its free-running oscillation, provided by a commercial RF source (Keysight E8257D) operating at 14.35 GHz with an output power of 0 dBm. In contrast to the conventional injection-locked OEO, where the frequency of the external RF signal matches the OEO’s generated signal frequency corresponding to the difference between the optical carrier and a single sideband, our second-order subharmonic injection-locked OEO operates with the external RF signal at a fractional subharmonic frequency – specifically, 1/2 of the OEO’s generated signal frequency. This approach effectively reduces the frequency of the external signal, resulting in lower system cost and power consumption. Figure 4a presents the measured optical spectrum, which includes both the first-order modulation sidebands and the residual optical carrier. The 14 dB lower power of the carrier than the first-order modulation sidebands does not affect the frequency of the generated oscillation.

Figure 4b displays the RF spectrum of the OEO captured by an RF spectrum analyzer (RFSA) with a frequency span of 600 kHz and a resolution bandwidth (RBW) of 2.4 kHz, which clearly indicates a central oscillation frequency at 28.7 GHz. The SMSR reaches 80 dB, representing an enhancement of 66 dB over the free-running OEO. The inset shows the long-term frequency stability of the RF signal, measured using the Maxhold function of a RFSA with a frequency span of 60 Hz and RBW of 1 Hz. The linewidth increased from 1 Hz to 13.7 Hz over 13 min, corresponding to a relative frequency drift of 5.67 × 10^−13^ per second. This instability is primarily due to misalignment of the optical carrier with the center of two adjacent MRR resonance peaks, unaddressed temperature and vibration effects on the fiber, and coupling jitter between the fiber and chip, resulting in unlocked state after 13 min. Future improvements will focus on stabilizing the MRR resonance, isolating the fiber, and packaging the TFLN photonic chip to enhance long-term frequency stability.

The comparison of phase noise for the 28.7 GHz injection RF signal – generated by the free-running OEO (black curve), the second-order subharmonic injection-locked OEO (red curve), and the Keysight E8257D (blue curve) – is illustrated in Figure 4c. For frequency offsets below 3 kHz from the 28.7 GHz center frequency, the injection-locked RF signal exhibits lower phase noise than the free-running OEO. At frequency offsets exceeding 3 kHz, the injection-locked OEO demonstrates superior performance, characterized by reduced phase noise and minimal spurious signals. Therefore, the locking range for the injection-locked OEO for an external RF signal with 0 dBm is estimated to be approximately 3 kHz, corresponding to the intersection of the phase noise curves for the injection RF signal and the free-running OEO. Within this range, phase noise is significantly suppressed compared to the free-running OEO; beyond it, the phase noise characteristic of the free-running OEO persists. At a 100 Hz offset from the 28.7 GHz center frequency, the measured phase noise is −81 dBc/Hz – a reduction of 51 dB relative to the free-running OEO. At a 10 kHz frequency offset, the injection-locked OEO achieves a phase noise of −117 dBc/Hz. Furthermore, spurious signals at integer multiples of 189 kHz, attributed to the OEO loop length of 1.058 km, are mitigated to levels below −107 dBc/Hz. These results demonstrate that the injection of an external 14.35 GHz RF signal enables the 28.7 GHz injection-locked OEO to simultaneously achieve ultra-low phase noise, high SMSR, and low spurious levels.

The locking range as a function of injection power is investigated by varying the injection signal power from −20 to 20 dBm, with results showing in the Figure 4d. The free-running OEO operates with RF signal frequency matching the MRR’s FSR. By injecting an external RF signal into the free-running OEO, when the RF signal frequency abruptly jumps to twice the injection frequency and the SMSR significantly improves, second-order subharmonic injection locking OEO realizes. The locking range is defined by recording the minimum and maximum injection frequencies, with the frequency differences multiplied by two representing the locking range. At an injection power of −20 dBm, the locking range is approximately 2.2 kHz, which represents the minimum input power required for injection locking. Below this power, the locked state is hard to achieve. As the input power is gradually increased to 20 dBm, the locking range reaches 162.2 kHz. The upper limit of 20 dBm is chosen because the maximum tolerable input power for our TFLN MZM is approximately 20 dBm. To ensure device safety, the injection signal power is not increased beyond this level; thus, 20 dBm is considered the maximum allowable injection signal power. It can be seen that when the injection signal power is 0 dBm, the locking range in Figure 4d is 22 kHz, which differs from the 3 kHz in Figure 4c. This is due to environmental instability, which causes a decrease in the free-running OEO signal power. According to the relationship between locking range and signal power [24] (see Supplementary Material, Eq. (4)), the locking range generally increases with decreasing signal power. As the test duration increases, the gradual decrease in power leads to a steady rise in the locking range. Therefore, the measured locking range of 22 kHz at 0 dBm in Figure 4d is larger than the locking range of 3 kHz in Figure 4c.

The 28.7 GHz free-running OEO is subsequently injection locked using the sixth-order subharmonic of the free-running signal, sourced from a commercial RF generator (Keysight E8257D) operating at 4.78 GHz with an output power of 15 dBm. In contrast to the 0 dBm injection level employed for second-order subharmonic injection-locked OEOs, the greater injected power in this context compensates for the lower power of third-order modulation sidebands used in generating the desired RF signal [32].

**Figure 5:**
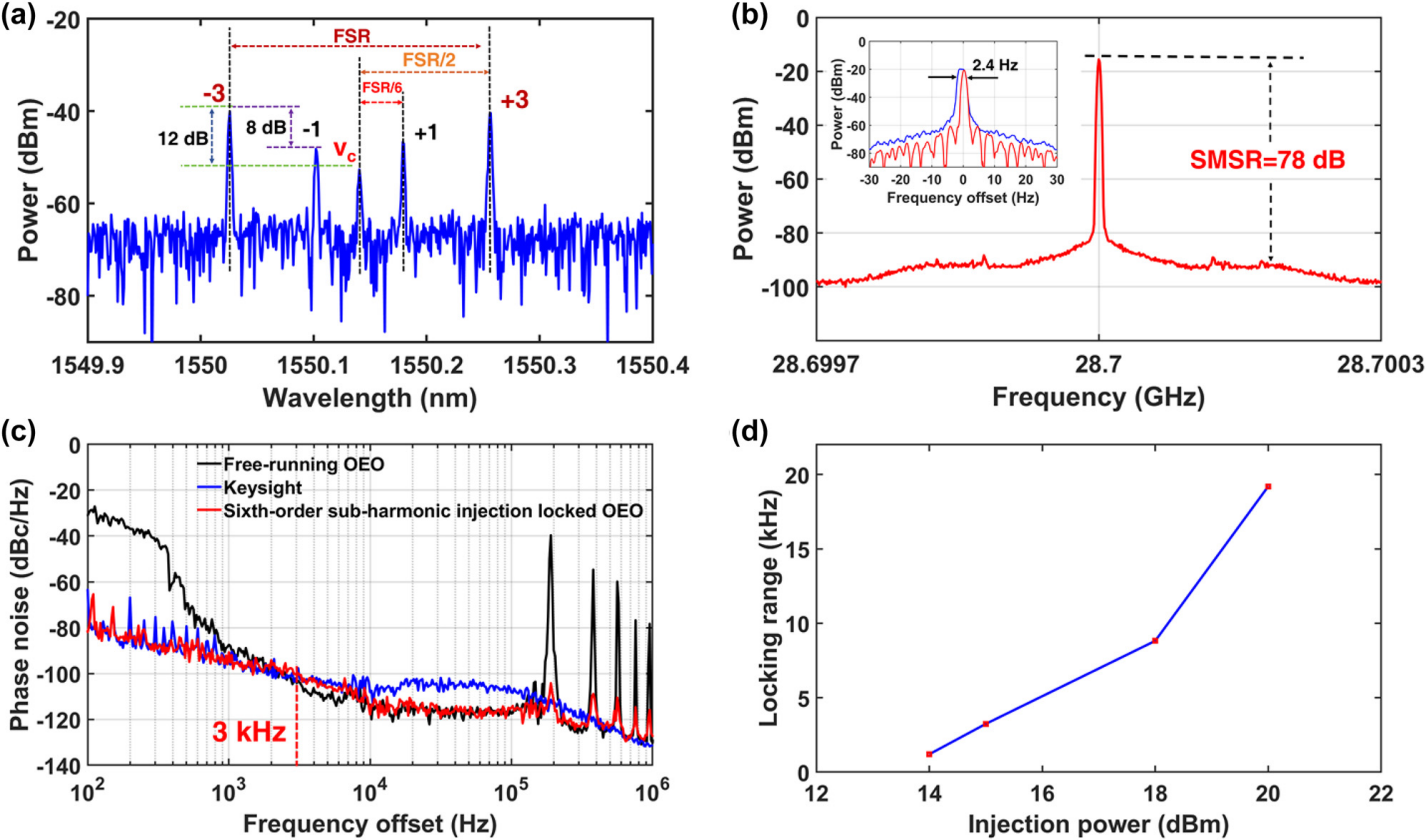
Sixth-order subharmonic injection-locked OEO. (a) The measured optical spectrum. (b) The RF spectrum of the 28.7 GHz signal measured with an RFSA with a frequency span of 600 kHz and a RBW of 2.4 kHz. Inset: The RF spectrum of the sixth-order subharmonic injection-locked OEO measured with an RFSA at Maxhold mode (blue curve) within 2 min and normal mode (red curve). (c) The measured phase noises of the 28.7 GHz RF signal generated by the free-running OEO (black curve), the sixth-order subharmonic injection-locked OEO (red curve), and the external injected RF source (Keysight E8257D) (blue curve). (d) The measured locking range as a function of the injection power.

Figure 5a presents the measured optical spectrum, which reveals the presence of third-order modulation sidebands along with residual first-order sidebands and the optical carrier. Notably, only the third-order modulation sidebands are selected by two resonance peaks of the MRR and subsequently directed to the PD for beating to generate the OEO oscillation, as illustrated in Figure 5b. The SMSR is measured at 78 dB, representing an improvement of 64 dB compared to the free-running OEO. The inset shows the long-term frequency stability of the RF signal, measured using the Maxhold function of a RFSA with a frequency span of 60 Hz and RBW of 1 Hz. The linewidth increased from 1 Hz to 2.4 Hz over 2 min, corresponding to a relative frequency drift of 4.07 × 10^−13^ per second.

Figure 5c compares the phase noise profiles of the 28.7 GHz RF signals generated by the free-running OEO (black curve), the sixth-order subharmonic injection-locked OEO (red curve), and the Keysight E8257D (blue curve). The results indicate that the phase noise of the locked RF signal closely aligns with that of the commercial RF source (KEYSIGHT: E8257D) for frequency offsets below 3 kHz from the 28.7 GHz carrier, while matching the characteristics of the free-running OEO at offsets beyond 3 kHz. Additionally, undesired spurious signals at integer multiples of 189 kHz are significantly mitigated by the proposed device.

Figure 5d presents the locking range of the sixth-order subharmonic injection-locked OEO as the injection signal power increases from 14 dBm to 20 dBm. The minimum input power for injection locking at 14 dBm is significantly higher than the minimum input power of −20 dBm for second-order subharmonic injection locking OEO. This is due to the significantly lower ±3rd-order sidebands power generated by the MZM compared to the ±1st-order sidebands. The injection signal power is also limited to a maximum of 20 dBm to ensure device safety as previously discussed.

By injecting an external RF signal at 4.78 GHz, the system achieves a high-frequency 28.7 GHz RF output characterized by low phase noise and minimal spurs. We experimentally attempted to generate a 28.7 GHz signal by injecting an external RF signal at 2.87 GHz, but failed. Subsequent simulations revealed that the injection locking using the tenth-order subharmonic of the free-running signal with power of 20 dBm requires that the modulator’s *V*_*π*_ should be reduced to a value of 1.4 V to produce sufficiently high-power modulation sidebands. Furthermore, integrating a TFLN MZM with lower *V*_*π*_ and increased EO bandwidth could facilitate realization of higher-order subharmonic injection-locked OEOs, leveraging compact, low-frequency, high-performance crystal oscillators to generate even higher-frequency signals. Looking forward, an electro-optic frequency comb will be employed to generate the requisite high-power modulation sidebands, which is expected to enable the implementation of OEOs based on higher-order subharmonic injection locking.

A detailed comparison of the second-order and sixth-order subharmonic injection-locked OEOs is presented in Table 1, covering the injection signal frequency, power, SMSR, phase noise, long-term frequency stability, and locking range. The sixth-order scheme generates the same output frequency from a lower-frequency injection signal, which relaxes the demands on the external source, thereby reducing system cost and power consumption. However, its operation relies on the ±3rd-order modulation sidebands for frequency beating, necessitating a higher injection power under the same *V*_*π*_ to achieve locking. Aside from these differences, Table 1 shows that the two OEOs achieve comparable performance in SMSR, long-term frequency stability, phase noise, and locking range.

**Table 1:** Comparison of second-order and sixth-order subharmonic injection locking TFLN OEO.

	Injection signal frequency (GHz)	Injection signal power (dBm)	SMSR (dB)	Relative frequency shift (/s)	Phase noise @100 Hz/@10 kHz (dBc/Hz)	Locking range (kHz)
Second-order	14.35	0	80	5.67 × 10^−13^	−82/−117	3
Sixth-order	4.78	15	78	4.07 × 10^−13^	−81/−118	3

## 6 Discussion and conclusion

In summary, a subharmonic injection-locked photonic integrated OEO on a TFLN platform is experimentally demonstrated using a TFLN PIC composed of an MZM and an add-drop MRR. The MZM is constructed with a folded CL-TWE design, featuring a total modulation length of 2.1 cm, which achieves a measured *V*_*π*_ of 1.7 V and a 3 dB EO bandwidth of 38 GHz. This *V*_*π*_ is lower than typical values of over 4 V for conventional MZMs fabricated with bulk LN [27], [28], enabling the generation of higher-order modulation sidebands with greater power so that a lower frequency RF signal can be injected to obtain a higher frequency RF output. The FSR of the add-drop MRR is set at 28.7 GHz, supporting OEO oscillation at this frequency and filtering out other oscillation modes. The second-order subharmonic injection-locked photonic integrated OEO is demonstrated by injecting a 14.35 GHz RF signal, producing a signal at 28.7 GHz, an SMSR of 80 dB, and phase noise of −81 dBc/Hz at 100 Hz and −118 dBc/Hz at 10 kHz frequency offsets from the carrier. Additionally, sixth-order subharmonic injection-locked photonic integrated OEOs are shown by injecting a 4.78 GHz RF signal, with a generated frequency of 28.7 GHz, an SMSR of 78 dB, and phase noise of −82 dBc/Hz at 100 Hz and −117 dBc/Hz at 10 kHz offsets. For comparison, the free-running OEO using a 1-km long SMF has an SMSR of 14 dB and phase noise of −30 dBc/Hz@100 Hz; the device presented here shows an SMSR improvement of more than 60 dB and a notable reduction in phase noise by 50 dBc/Hz@100 Hz. In both high-order subharmonic injection-locked photonic integrated OEOs, the spur can be reduced to −105 dBc/Hz, demonstrating the device’s spur suppression capability.

A comparison with previously reported integrated OEOs in recent years is presented in Table 2, which shows that the near-carrier phase noise of the injection-locked TFLN OEO is lower and the SMSR is higher.

**Table 2:** Comparison of our work with the previous integrated OEOs.

Scheme	Integrated components	Fiber length (km)	*f*_Inj_ (GHz)	*f*_out_ (GHz)	SMSR (dB)	Phase noise @100 Hz/@10 kHz (dBc/Hz)
SOI [4]	PM, MDR, PD	–	–	3–7.4	67	−40/−80
SOI [5]	Hybrid-integrated	0.5	–	3–18	70	−38/−116
SOI [6]	MZI, MRR, PD	0.05	–	0–20	45	−30/−80
InP [7]	DML, ODL, PD	–	–	8.87	25	−50/−60
SiN-LN [8]	PM, MRR	0.0468	–	3–42.5	48	−20/−93
TFLN [9]	MZM, MRR	0.025	–	30	50	−40/−102
TFLN [9]	PM, MRR	0.025	–	20–35	49	−38/−87
TFLN [10]	MZM, MRR, MZI	0.025	–	20–35	47	−40/−85
**This work**	**MZM, MRR**	**1**	**14.35**	**28.7**	**80**	**−82/−117**
		**1**	**4.78**	**28.7**	**78**	**−81/−118**

Using this device, phase noise of RF signals at frequency offsets close to the carrier frequency can be adjusted by employing an external commercial low phase noise RF source, while for larger offsets, phase noise depends on the characteristics of the signal generated by the free-running OEO. This approach supports the generation of integrated high-frequency RF signals with low phase noise, high SMSR, and low spurs concurrently. If a TFLN MZM with reduced V_π_ and increased EO bandwidth is developed, it becomes possible to substitute the RF source with a compact, cost-effective crystal oscillator, eliminating the need for higher frequency RF sources. This will be more beneficial for achieving a compact OEO, reducing system cost and power consumption. Additionally, the integration of semiconductor lasers and PD on the TFLN platform [33], [34], [35] may facilitate the heterogeneous integration of all photonic components onto a single chip.

## 7 Materials and methods

TFLN PIC fabrication: The PIC for the subharmonic injection-locked OEO was engineered and manufactured on an X-cut TFLN platform featuring a 360 nm device layer. Initially, all optical waveguides were defined using electron beam lithography (EBL) followed by inductively coupled plasma (ICP) etching. A 1 µm-thick silicon dioxide (SiO2) layer was then deposited via plasma-enhanced chemical vapor deposition (PECVD). Nickel-chromium (NiCr) loads with a thickness of 0.2 µm and an additional gold layer of 0.9 µm were fabricated through electron beam evaporation. The capacitance-loaded traveling-wave electrodes (CL-TWEs) were produced using a lift-off process. In this context, CL-TWEs [36] with air bridges were incorporated into TFLN modulators to minimize the geometric length of the device while maintaining a low driving voltage. The silicon oxide layers above and below the electrodes both measured 1 µm in thickness.

MRR design: To achieve MRR with superior performance, the width of the ring waveguide, bus waveguide, and the gap between them are 2.2 µm, 1.376 µm, and 0.8 µm, respectively.

## Supplementary Material

Supplementary Material Details
