# Supplementary material for: Subharmonic injection-locked photonic integrated thin-film lithium niobate optoelectronic oscillator
Source: Nanophotonics. 2025 Nov 19;14(26):4793–803. doi: 10.1515/nanoph-2025-0476 (PMC12714060; doi:10.1515/nanoph-2025-0476)
Supplement: Supplementary file 1 — Supplementary Material Details [file j_nanoph-2025-0476_suppl_001.docx]

Supplementary Information to:

Zijun Huang^1,#^, Rui Ma ^1,#^, Qiang Ying^1^, X. Steve Yao^2,3,*^, Peng Hao^2,*^, Wei Ke^1^, Xinlun Cai^1,4,*^

**Sub-harmonic injection-locked photonic integrated Thin Film Lithium Niobate Optoelectronic Oscillator**

^1^State Key Laboratory of Optoelectronic Materials and Technologies, School of Electronics and Information Technology, Sun Yat-sen University, Guangzhou 510275, China

^2^Photonics Information Innovation Center and Hebei Provincial Center for Optical Sensing, College of Physics Science and Technology, Hebei University, Baoding 071002, China

^3^NuVison Photonics, Inc, Las Vegas, NV 89109, USA

^4^Hefei National Laboratory, Hefei 230088, China

*Corresponding author: [syao@ieee.org](mailto:syao@ieee.org), [haopeng@hbu.edu.cn](mailto:haopeng@hbu.edu.cn), [caixlun5@mail.sysu.edu.cn](mailto:caixlun5@mail.sysu.edu.cn)

*#These authors contributed equally to this work.*

**1 Locking range of injection locked OEO**

The locking range of the injection-locked OEO is analyzed using its governing differential equation [1], [2], which expresses the OEO phase as a function of time,

|  |  | (1) |
| --- | --- | --- |

 and $\Delta\omega_{0}$ are the phase difference and the angular frequency difference between the free-running OEO signal and the injection signal, respectively, $Q$ is the quality factor of the OEO loop, $E_{inj}$ and $E_{osc}$ are the voltage of the injection signal and the free-running signal, respectively.

In the steady state, the left-hand side of Eq. (1) equals zero, thus we have

|  |  | (2) |
| --- | --- | --- |

The locking range of the injection-locked OEO is found by solving Eq. (2) for a maximum, which occurs when = 1, and thus the locking range is given by

|  | $\Delta\omega_{0,\max}=\frac{\omega_{0}}{2Q}\frac{E_{inj}}{E_{osc}}$ | (3) |
| --- | --- | --- |

By substituting $Q=\omega_{0}T$ and $f_{FSR}=1/T$ into Eq. (3), we have

|  | $\Delta\omega_{0,\max}=\frac{f_{FSR}}{2}\frac{E_{inj}}{E_{osc}}$ | (4) |
| --- | --- | --- |

where $f_{FSR}$ is the mode spacing of the OEO, which is determined by the loop length. According to Eq. (4), the locking range of the injection-locked OEO is determined by the mode spacing of the OEO and the voltage ratio of the injection signal to free-running signal.

**2 Experimental setup for measuring the frequency response of the SSOL gain of the OEO**

Fig.S1 shows the experimental setup for measuring the SSOL gain of the OEO. A VNA (Agilent Technologies: N5227A) is placed between the FD and the EC to characterize the frequency response of the SSOL gain by measuring the transmission coefficient S_21_. A scanning RF signal generated from port 1 of the VNA is injected into the FD for frequency division before being fed into the MZM to modulate the optical carrier with a wavelength of 1550.14 nm and a power of 18 dBm. By thermally-tuning the MRR, the optical carrier is set at the center of two adjacent resonances of the MRR, with its modulation sidebands scanning on both sides of the optical carrier. Serving as a comb filter, the add-drop MRR allows only those modulation sidebands that coincide with the two adjacent transmission peaks to pass through with minimal loss; these are subsequently amplified by an erbium-doped optical fiber amplifier (EDFA) to offset optical losses. The modulation sidebands are finally converted back into RF signal by beating in the PD and being amplified by the LNA, before sending back to port 2 of the VNA.


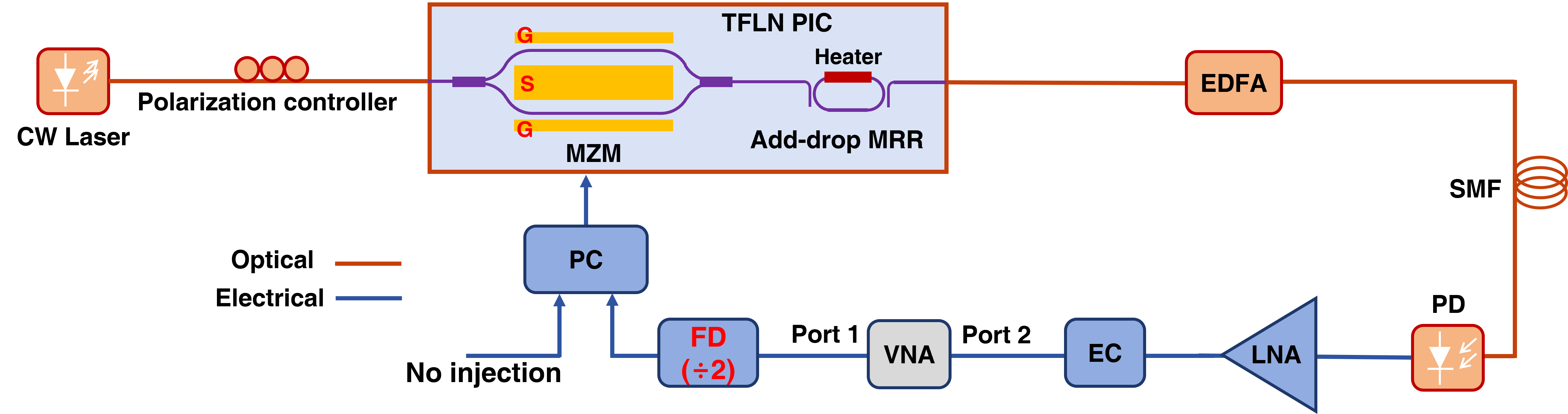


**Fig. S1.** Experimental setup for measuring the SSOL gain of the OEO. CW laser: continue wave laser; TFLN PIC: thin-film lithium niobate photonic integrated chip; MZM: Mach-Zehnder modulator; EDFA: erbium doped fiber amplifier; SMF: single mode fiber; PD: photodetector; LNA: low noise amplifier; VNA: vector network analyzer; PC: power combiner.

**Supplementary References**

[1] R. Adler, “A study of locking phenomena in oscillators,” Proceedings of the IEEE, vol. 61, no. 10, pp. 1380–1385, 1973, <https://doi.org/10.1109/PROC.1973.9292>.

[2] Z. Fan *et al.*, “Injection locking and pulling phenomena in an optoelectronic oscillator,” *Opt. Express*, vol. 29, no. 3, p. 4681, 2021, <https://doi.org/10.1364/OE.416253>.
